# B-assembler: a circular bacterial genome assembler

**DOI:** 10.1186/s12864-022-08577-7

**Published:** 2022-05-11

**Authors:** Fengyuan Huang, Li Xiao, Min Gao, Ethan J. Vallely, Kevin Dybvig, T. Prescott Atkinson, Ken B. Waites, Zechen Chong

**Affiliations:** 1 Informatics Institute, Heersink School of Medicine, the University of Alabama at Birmingham, AL 35294 Birmingham, USA; 2 Department of Genetics, Heersink School of Medicine, the University of Alabama at Birmingham, AL 35294 Birmingham, USA; 3 Department of Medicine, Heersink School of Medicine, the University of Alabama at Birmingham, AB 35294 Birmingham, USA; 4 Department of Pediatrics, Heersink School of Medicine, the University of Alabama at Birmingham, AL 35233 Birmingham, USA; 5 Department of Pathology, Heersink School of Medicine, the University of Alabama at Birmingham, AL 35233 Birmingham, USA

**Keywords:** Bacteria genome, De novo assembly, Long-read-only assembly, Hybrid-read assembly

## Abstract

**Background:**

Accurate bacteria genome de novo assembly is fundamental to understand the evolution and pathogenesis of new bacteria species. The advent and popularity of Third-Generation Sequencing (TGS) enables assembly of bacteria genomes at an unprecedented speed. However, most current TGS assemblers were specifically designed for human or other species that do not have a circular genome. Besides, the repetitive DNA fragments in many bacterial genomes plus the high error rate of long sequencing data make it still very challenging to accurately assemble their genomes even with a relatively small genome size. Therefore, there is an urgent need for the development of an optimized method to address these issues.

**Results:**

We developed B-assembler, which is capable of assembling bacterial genomes when there are only long reads or a combination of short and long reads. B-assembler takes advantage of the structural resolving power of long reads and the accuracy of short reads if applicable. It first selects and corrects the ultra-long reads to get an initial contig. Then, it collects the reads overlapping with the ends of the initial contig. This two-round assembling procedure along with optimized error correction enables a high-confidence and circularized genome assembly. Benchmarked on both synthetic and real sequencing data of several species of bacterium, the results show that both long-read-only and hybrid-read modes can accurately assemble circular bacterial genomes free of structural errors and have fewer small errors compared to other assemblers.

**Conclusions:**

B-assembler provides a better solution to bacterial genome assembly, which will facilitate downstream bacterial genome analysis.

**Supplementary Information:**

The online version contains supplementary material available at 10.1186/s12864-022-08577-7.

## Background

Genome assembly is the basis and prerequisite for understanding the genomic and functional characterization of organisms. There are approximately 5 × 10^30^ bacteria on the earth, the number of which exceeds all plants and animals [1], and a considerable number of them play an important role in the human microbiome [2]. For example, some bacteria that colonize the gastrointestinal tract are beneficial [3], while others are pathological and induce infectious diseases and antibiotic resistance [4]. Determining the genome sequences of bacteria is critical to conduct human microbiome associated health studies. However, to date, there are only a small number of bacterial genomes which have been published, and most of the published genomes are incomplete. For example, 90% of bacterial genomes in GenBank [5, 6] are incomplete. Incomplete bacteria genomes will make it challenging to conduct subsequent genomic analyses, such as genome structure, genome annotation, variant discovery, comparative genomics, etc. Thanks to Next-Generation Sequencing (NGS) technologies, sequencing data and published bacterial draft genomes have grown dramatically [5]. Nevertheless, bacterial genomes can contain up to several dozens of repetitive sequences, which may be much longer than the maximum read length and the insert size of paired-end tags [7]. Such regions are challenging for bacterial genome assembly as they may lead to genome sequences with dozens of fragmented or wrong assemblies [8].

With the advent of third generation sequencing platforms such as Pacific Biosciences (PacBio) and Oxford Nanopore Technologies (ONT), which can read complete DNA fragments of 10 kbp or longer and can cover tandem repeats [8–10], there is great potential to improve the quality of bacterial genome assemblies. However, the existing DNA assemblers are far from optimal for this purpose for the following reasons. First, like eukaryotic genomes, bacterial genomes can also have long and high density of repetitive sequences [11, 12]. PacBio or ONT reads cannot fully cover these regions, which can lead to fragmentary or incorrect assemblies. Second, ONT or PacBio reads have much higher error rates (indels and base errors) [13] that may cause low-quality assemblies and lead to false annotations thereafter. Although there are polishing tools (i.e., pilon and Racon) [14, 15] that can address this problem to some extent, after polishing, the contigs constructed from error-prone long reads will still have errors [16]. Finally, most of bacterial genomes consist of a single DNA molecule (i.e., one chromosome) that is several million base pairs in size and is circular. However, existing long-read assemblers like Canu [17], wtdbg2 [18], and Flye [19], etc. are not specifically designed for bacterial genomes as they are not aware of the circular structures.

To our knowledge, there are few long-read assemblers that can generate high-quality bacterial genome assemblies. Unicycler [20] is one of most frequently used tools for bacterial genome assembly, and it has three modes of input: long-read-only, Illumina-only, and hybrid reads. Therefore, it accepts PacBio, ONT, Illumina data, or a combination of them. Unlike linear genome assemblers, Unicycler can circularize replicons without the need of postprocessing of assembly results. Additionally, in the hybrid mode, it needs as low as 10 × long read sequencing data. However, in the long-read-only mode, Unicycler uses minimap and miniasm [21] to assemble the long reads, which will generate contigs with a similar error rate to raw long reads, and the contigs that it produces tend to collapse repeats or segmental duplications. Although Unicycler uses Racon [15] to improve the accuracy of contigs, it cannot alleviate the structural errors caused by repeat/duplication collapses. In the hybrid-read mode, Unicycler first builds a graph with Illumina short reads by using the short-read assembler SPAdes [22], then it creates bridges with long reads in order to resolve the repeats in the genome. Even so, starting from short-read assemblies may lead to many structural errors due to the presence of repeats that are longer than the short-read lengths. Moreover, mapping long, noisy reads to short read assembled contigs is challenging because the contig lengths are relatively short, and they often contain structural errors. For a successful bridging, several high-quality and *bona fide* alignments that can cover the unsolved repeats as well as a large portion of their flanking regions are required. Therefore, it is hard, if not impossible, to solve large repeats. As a result, Unicycler is more likely to create fragmented assemblies or wrong assemblies with many structural errors instead of a complete genome. Thus, it is necessary to develop an alternative assembler which can address these issues.

### Implementation

In order to generate higher quality assemblies, we present a new method, B-assembler, for bacterial genome *de novo* assembly. B-assembler accepts hybrid reads from both long and short reads and long-read-only data. B-assembler consists of three main steps: constructing the initial genome, reassembling the boundaries of the genome, and forming the circular genome. B-assembler applies the long reads method first, and then corrects the long noisy reads using Racon [15] before assembly in order to minimize ambiguities for finding overlapping sequences. It uses Flye [19] as the core assembler for both assembly modes. If short reads are also provided, the short reads will be used to polish long reads and the final assembly. Compared with other assemblers, B-assembler has several advantages. First, B-assembler can reconstruct circular bacterial genomes, while other genome assemblers except Unicycler cannot achieve this goal for bacterial genome assembly by design. Second, it partitions the long reads of varied lengths into two groups: longer and shorter. By starting from the longer reads group, B-assembler can achieve accurate assembly results which are nearly free of structural errors and have few base errors. Lastly, compared with the other hybrid assemblers and hybrid-read mode of Unicycler, B-assembler has a shorter runtime and requires less memory usage. Therefore, B-assembler can obtain high-quality assemblies from long noisy reads, which are critical for downstream genomic analysis of microbes.

### Long-read-only assembly

Long-read only mode accepts either ONT or Pacbio raw reads as input. It contains three main steps: creating the initial assembly, reassembling assembly ends, and merging contigs to construct a circular genome.

#### Initial assembly

Long reads make genome assembly easier and provide the possibility to resolve repeats and structural variants that are several kilobases in length. To eliminate the possibility of generating incomplete genome, B-assembler calculates the total length and coverage of all long reads and separate them into two subsets by their length. One subset ($S1$) consists of the longest reads which have coverage over 50X (see Supplementary Table 1). The other subset ($S2$) contains all the remaining reads. Before constructing initial assembly, it aligns $S2$ to $S1$ using minimap2 with the parameter of ‘-ax map-ont’ or ‘-ax map-pb’. The alignments are then used as input to correct reads by Racon. By performing this step, $S1$ long reads with a high error rate will be corrected to some extent, which will improve the accuracy of genome assembly. After $S1$ correction, B-assembler applies Flye to construct an initial assembly ($L1$).

#### Reassembly of the ends

Since Flye cannot resolve circular bacterial genomes and therefore tends to introduce errors at the two ends of assemblies, B-assembler reassembles the two ends to address this issue. First, it aligns all the long reads back to $L1$ using minimap2 and then, it extracts the reads aligned to the two ends. To identify error-rich regions, we have also plotted the error frequencies of $L1$ from several nanopore sequencing data and found that the errors mainly gathered in the first and last 20% of $L1$ (data not shown). Therefore, only reads that were mapped to the first and last 20% of $L1$ are retained, which are called end-reads. In addition, only the end-reads that have high mapping quality ($\ge$ 20) are used for reassembly. Then the end-reads are assembled into a secondary assembly. Let the secondary assembly be $L2$. Before merging $L1$ and $L2$, $L2$ will be polished to mitigate base errors rate. Therefore, $L2$ goes through two correction processes with mapped reads using Racon.

#### Generating the circular genome

Most bacteria have a genome that consists of a single DNA molecule (i.e., one chromosome) that is several million base pairs in size and is "circular" (doesn't have telomeres like eukaryotic chromosomes). B-assembler performs several additional steps instead of directly merging two rounds of assemblies to achieve a circular genome. Basically, it removes the overlapping sequences and joins the unique sequence to form a circular genome. The details of the process are as follows: B-assembler applies minimap2 to align $L1$ and $L2$ with ‘-cx asm20’ parameter to identify overlapping and unique sequences. It then discards 40% of overlapped $L2$ sequences (20% of each side) and keeps the middle part (60% of length) of $L2$. The discarded sequences will be substituted by the overlapping sequences of $L1$. Therefore, the final whole genome sequence consists of 60% of the $L2$ (middle part), 40% of the overlapped sequences, and the unique sequences of the $L1$. Let overlapped and unique sequences in $L1$ be $O1$ and $U1$, respectively, overlapped and unique sequences in $L2$ be $O2$ and $U2$, and new merged assembly be $A$, then:

$$A=\left(L2-L2*40\%+U2\right)+(U1+O1*40\%)$$

#### Recognition of the start position and performing the final correction

The starting position of the assembled circular sequence can be located at any site if not considering the bacterial genome structures. To lay out the circular genome starting from the conventional starting point, *dnaA*, a gene is found in most bacteria and usually close to the origin of replication [23], B-assembler uses TBLASTN to search for the *dnaA* alleles. If hits are found, the sequence will be shifted based on the best hit so that the genome begins with the *dnaA* gene and on the forward strand. If no such matches are found, the sequence will keep the default starting point.

As a final step, B-assembler uses minimap2 to map the reads to the final assembly and uses Flye’s ‘–polish-target’ function to polish the assembly for long-read-only mode.

### Hybrid assembly

The method of hybrid-read mode contains the three key steps of the long-read-only mode, including 1) initial assembly, 2) reassembly using the end-reads, and 3) merging the initial and reassembled contigs into a complete, circular genome. The difference between long-read-only and hybrid modes is that since the Illumina reads have higher accuracy, B-assembler takes advantage of short reads instead of long reads for polishing and therefore can achieve more accurate assembly results. It uses short reads for the following two steps: 1) Before initial assembly, B-assembler uses bwa mem [24] to align short reads to all long reads. The alignments are then used for correction by Racon with ‘-e 0.1 -m 10 -q 30’. Then the corrected long reads are the input for the key three steps. 2) At the final step, it uses short reads to polish the circular contig. By comparing the performances of several existed short-read polishing tools apollo (v2.4.0) [25], racon (v1.4.20) [15] pilon (v1.23) [14] and NextPolish (v1.3.1) [26] (see Supplementary Table 2), we selected pilon for the final polishing in hybrid mode. It uses bwa mem to map short reads back to circular contigs, and pilon with –fix all to polish. The polishing procedure repeats three times to achieve a whole genome sequence with the minimal errors.

### Benchmarked datasets

For Simulation data, we simulated both long and short reads from one mycoplasma species *M. arginini* [27]. It has been detected in both healthy and diseased animal hosts (mouse, goat, monkey, etc.) and is generally considered a colonizer in animals [28, 29]. Even though the size of the *M. arginini* genome is small (678,592 bp), it contains pervasive tandem repeats, which create a challenge for effective genome assembly. The reference genome used for the simulation was the strain HAZ 145_1 [27] downloaded from the NCBI database. ONT reads were simulated using NanoSim (v2.4-beta) [30], an ONT read simulator designed to generate artificial long reads. The total number of simulated ONT reads was 20,798 and covers 300X of the genome, and the length was set in the range of 0.1-130kbp with a mean of 10kbp. We also generated a synthetic paired-end short reads dataset for the same species using ART version 2.5.8 [31]. The simulated short reads mimic those from an Illumina Miseq v3: 2 × 250 bp pair-end reads, 300 bp mean insert size, 10 bp insert size standard deviation, and 250X read depth.

For Nanopore sequencing data, the real sequences were from two clinical isolates of mycoplasma species, *M. arginini* (strain 51,226) and *M. amphoriforme* (strain 69,156). For *M. arginini*, both high-coverage ONT long reads (208X) and Illumina short reads (7900 X) were obtained. *For M. amphoriforme,* only ~ 364 X ONT long reads were sequenced. All the ONT reads were sequenced by a MinIon sequencer. ONT library was prepared using a Rapid Sequencing Kit (SQK-RAD004) and run on a MinION Flow Cell (R9.4). Illumina reads were sequenced by Illumina MiSeq platform in the UAB Heflin Genomic Core.

For PacBio sequencing data, we downloaded 14 bacterial strains [32] which included both Gram-positive and -negative species from the National Collection of Type Cultures (NCTC) 3000 project on the basis of there being high-quality reference genome sequences of the same strains available for comparison (see Additional file 1 Table S5 for species and reference genome accession numbers).

### Benchmarked tools

As a comparison, these data were also run on several other popular assemblers, including wtdbg2 (v2.5) [18], Flye (v2.7.1) [19], Canu (v1.8) [17], apollo (v2.4.0) [25], racon (v1.4.20) [15] pilon (v1.23) [14], NextPolish (v1.3.1) [26], Unicycler (v0.4.8) [20] long-read-mode, and Unicycler hybrid-mode. All the algorithms were run under the default settings or recommended settings based on their manuals. All the tools were tested on Cheaha, the High-Performance Computer Server of the University of Alabama. The server is × 86-64bit based Linux system. All the tasks were submitted via Slurm job scheduling system with 4 CPUs and a total of 60 GB memory.

### Assembly evaluation

For the genomes with a complete reference sequence (simulation data and PacBio sequencing data), we applied QUAST (v4.3) [33] to calculate the assembly statistics for all the tested algorithms, including number of contigs, maximum contig length, genome fraction, GC content, number of misassemblies, number of local misassemblies, duplication ratio, number of mismatches per 100kbp, and number of indels per 100kbp. We also used Mauve [34] to visualize the alignments between individual assembly vs. the reference.

For the real nanopore data, we inspected read alignments with respect to the contigs to assess the assemblies’ structural accuracy. Since the bacterial genomes are haploid, the supplementary alignments cluster in one region are unexpected, indicating a potential structural assembly error. By aligning the nanopore reads back to the assembly using minimap2, the Supplementary alignments were extracted using samtools. We defined a supplementary cluster as more than 10 supplementary alignments enriched in the same region. We included the metrics of total number of supplementary alignments and supplementary clusters for the benchmarked tools tested on the real ONT data.

Since the isolated *M. arginini* strain has both ONT and Illumina MiSeq data, these data were subjected to hybrid assemblies for B-assembler and Unicylcer. All the other real sequencing data were subjected to long-read-only *de novo* assembly.

### Experimental validation

To further evaluate the assembly results, 76 low-complexity regions of the *M. arginini* genome were selected for PCR amplification and Sanger sequencing validation. Minimap2 was used to map the PCR sequences to the assembled contigs. The mapping rate, single base pair mismatches, and indels were calculated to indicate the assembly accuracy.

## Results

### Workflow of B-assembler

**Fig. 1:**
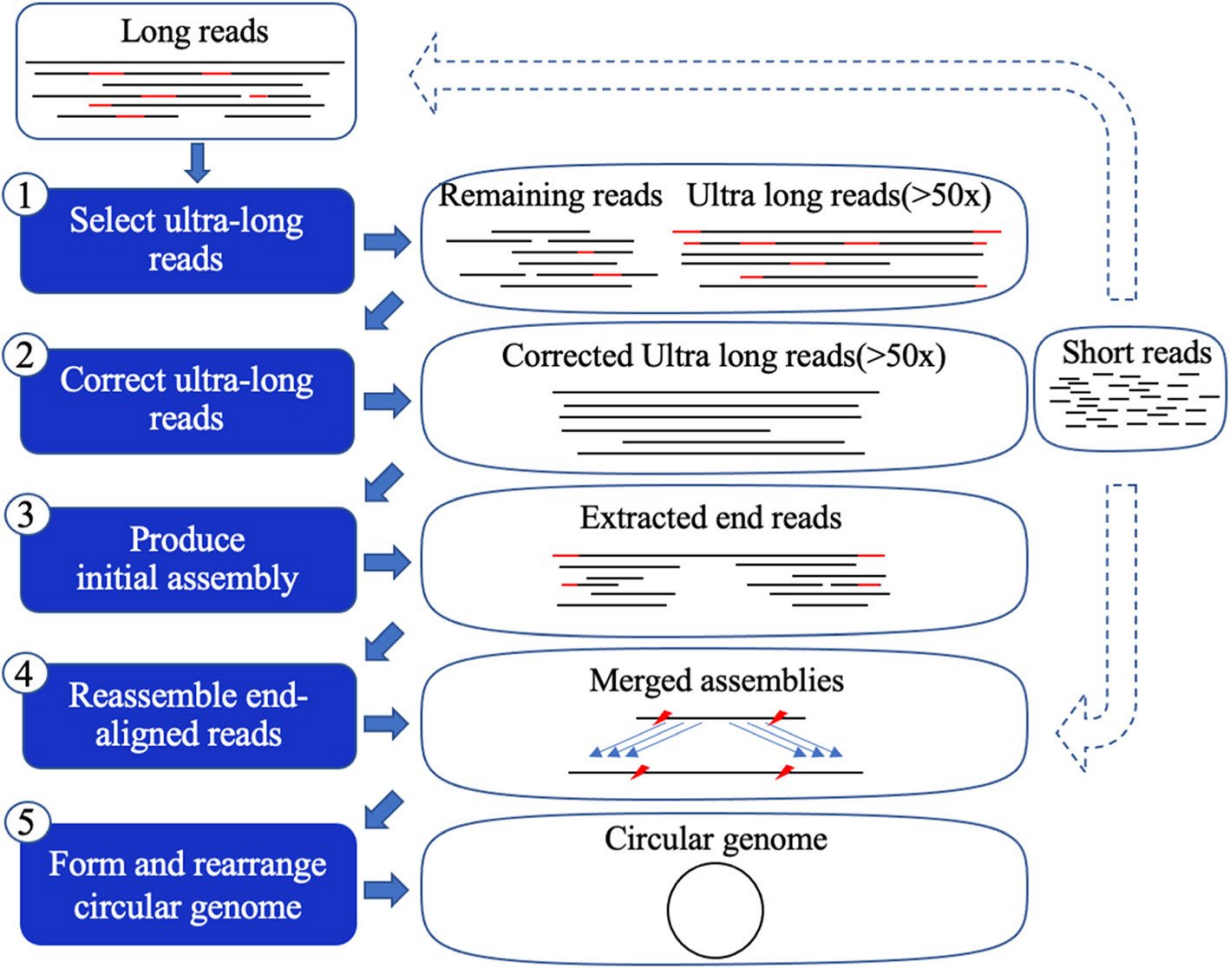
The workflow of B-assembler. B-assembler has two modes: long-read-only assembly and hybrid reads assembly

B-assembler is designed for ONT/Pacbio long-read only or hybrid reads (ONT/PacBio and Illumina) assembly (Fig. 1). In long-read only mode, it first selects the longest reads that cover ~ 50X (Additional file 1 Note 1 and Additional file 1 Table S1) of the genome and corrects the selected long reads by the remaining reads to produce an initial assembly. The two ends of the initial assembly undergo reassembly by a subset of reads that map to this region, and the contig generated by these reads replaces the two ends of initial assembly. When a join (merging of the two ends) is made, the complete and circular assembly is completed. Finally, B-assembler rearranges the start position of the merged assembly to the *dnaA* gene and applies a post-correction step that uses high-depth long reads to polish the circular draft assembly. In the hybrid-read mode, the key steps including assembling the corrected longest reads, reassembling the end reads, and forming the circular assembly are the same as the long-read only mode. In addition to this, it combines accurate short reads instead of long reads for correction processes (i.e., correcting all long reads and polishing final merged assembly). The hybrid mode provides a more optimized strategy for accurate bacterial genome assembly.

### Performance of B-assembler’s long-read-only mode on simulated ONT datasets

To evaluate the performance of B-assembler and make a comprehensive comparison with other assemblers, we first simulated long and short reads from a bacterial strain, *M. arginini* [27]. A total of 20,798 Nanopore reads were simulated at a depth of 300X using nanosim [30]. The read lengths range from 0.1kbp to 130kbp with a mean of 10kbp. Illumina 2 × 250 bp paired-end Miseq reads that covered ~ 250X of the genome were simulated using ART [31], with a mean insert size of 300 bp and 10 bp standard deviation. (Additional file 1 Note 2 and Additional file 1 Fig S1). In addition to B-assembler, we included long-read assemblers (wtdbg2, Flye, Canu) and hybrid assemblers (Unicycler, hybridSPAdes, haslr and lathe) for the comparison. We used the default parameters or recommended settings for all the tested tools. The performances of B-assembler and other assemblers were evaluated by QUAST assembly metrics [33].

For the long-read-only ONT dataset, QUAST statistics such as number of contigs, max contig length, genome fraction, etc. were recorded for all the benchmarked algorithms (Table 1). The full statistics were summarized in Additional file 1 Table S2. Although the *M. arginini* genome contains pervasive low-complexity sequences, all the assemblers can generate a single contig that closes to the reference size (678,592 bp) implying the advancement of these long-read assemblers. All assemblers (also shown in Additional file 1 Fig S2) except wtdbg2 had no misassemblies that represent large-scale structural errors (> 1000 bp) and no local misassemblies that are small-scale structural errors (> 85 bp and < 1000 bp). Wtdbg2 produced 1 misassembly and 4 local misassemblies. This is likely due to its homopolymer-compressed binning approach, which is less sensitive in repetitive regions, but wtdbg2 was the fastest algorithm and consumed the least memory.

**Table 1:** Evaluation of assembly on simulated sequences

Asm	# ctg	Largest contig	Cov %	# Local mis	# SNV	# Indels	Time(min)	Memory(G)
B-assembler L	1	668,045	98.446	0	0.9	0	56	8.9
wtdbg2	1	658,000	96.842	1	217.3	216.08	11	1.7
Flye	1	701,022	98.444	0	0.89	74.01	724	30.1
Canu	1	676,819	98.455	0	1.62	44.26	3,848	37.2
Unicycler L	1	680,945	99.839	0	65.47	225.6	70	3.5
B-assembler H	1	676,353	99.67	0	0	0	66	11.7
Unicycler H	1	677,975	99.809	0	7.48	5.15	1634	14.8
hybridSPAdes	2	433,245	99.947	1	6.78	0.88	42	16
haslr	1	613,987	90.47	0	1.79	13.36	4	1.3
lathe	1	673,506	95.49	1	12.48	26.75	85	6.7

*# Local mis* Local misasemblies, *Cov %* assembled genome fraction, *# SNVs* number of mismatches per 100kbp, *# Indels* number of Indels per 100kbp, *L* Long-read-only mode, *H* Hybrid mode

The reference genome has a GC content of 26.38%. Only B-assembler could achieve the exact GC value for the long-read only data. Although the genome fraction (percentage of aligned bases in the reference genome) of B-assembler ranked in the middle (98.446 vs. 99.839 of Unicycler’s long-read mode), the duplication ratio (the total number of aligned bases in the assembly divided by the total number of aligned bases in the reference genome) is 1. This implies, as stated in QUAST documentation, that the higher genome fraction of Unicycler and wtdbg2 may be due to contigs that mapped to multiple locations and thus counted multiple times.

The base accuracy was evaluated using number of mismatches and number of indels per 100kbp. Both B-assembler and Flye have the lowest number of mismatches (0.9 and 0.89, respectively). In contrast, Unicycler’s long-read-only mode and wtdbg2 have mismatches as high as 65.47 and 217.3 per 100kbp. B-assembler long-read-only mode uses Flye’s polishing module for the final polishing and therefore achieved almost the same substitution accuracy. While Unicycler’s used Racon and wtdbg2 introduced partial order alignment [35] to polish the assemblies, it seems there is still room to further improve the base accuracy. Interestingly, B-assembler had no indel errors, while Flye and the other assemblers high indel errors. This implies that the two-round genome assembly strategy works better than considering all reads as a whole to alleviate the indel errors.

Since only Unicycler and B-assembler are the circular-aware bacteria assemblers, it is not surprising to see that only these two software products can correctly identify the starting position of the assembled genome (Additional file 1 Fig S2).

In terms of runtime and peak memory usage, Canu spent over 68-fold time and ~ fourfold memory compared to B-assembler. Unicycler adopted miniasm [21] as its long-read assembly engine and it was faster and consumed less memory usage than B-assembler. However, as shown in Table 1, the assembly from Unicycler’s long-read-only mode contained too many errors. Interestingly, although B-assembler pipeline used Flye as the core assembly engine, B-assembler was faster and consumed less memory than Flye. Again, this may be due to B-assembler’s two-round assembly strategy which consider assembling subset reads instead of all reads, while Flye loaded all the reads at once to the main memory.

Considering all the evaluated factors, B-assembler surpassed the other benchmarked tools with the simulated long-read dataset and constructed the most accurate genome sequence.

### Performance of B-assembler’s hybrid-read mode on simulated datasets

By comparing several short reads polishing tools (apollo (v2.4.0) [25], racon (v1.4.20) [15] pilon (v1.23) [14] and NextPolish (v1.3.1) [26]) (See Additional file 1 Table S3), we selected pilon in B-assembler hybrid-read mode.

We compared B-assembler with Unicycler and other three hybrid assemblers (hybridSPAdes (v 3.15.2) [22], HASLR (v 0.8a1) [36] and lathe [37]) on the simulated ONT reads and Illumina reads. Except hybridSPAdes, B-assembler, Unicycler hybrid mode, haslr, and lathe all generated one complete contig. In.

addition, hybridSPAdes and lathe had one misassembly. For the base accuracy, B-assembler performed best since it can eliminate all mismatches and indels, while the other hybrid assemblers all induced some levels of mismatches or indels per 100kbp. Although HASLR was the fastest and consumed the least main memory, it can only cover 90.47% of the genome. The running time and memory usage of B-assembler was much lower than Unicycler. Therefore, B-assembler outperformed all the other hybrid assemblers overall.

In addition, to evaluate the resource usage from each component in the workflow, we stratified and benchmarked each key component of B-assembler pipeline and recorded the performance of each step. As shown in Additional file 1 Table S4, the numbers of mismatch and indel errors dropped as the pipeline ran. Thanks to the two-round assembly strategy and optimized parameters for the assembly and the error correction, a substantial drop was observed from the initial assembly to the second round of assembly to form and rearrange the circular genome.

### Performance of B-assembler’s long-read-only mode on real ONT datasets

To evaluate the performance of B-assembler on the real long-read-only dataset, we deep sequenced an *M. amphoriforme* strain isolated from an infected patient and generated 108 k reads covering ~ 305X of the genome, with over 94% of the reads are longer than 10kbp (Additional file 1 Fig S1). As a comparison, we also ran wtdbg2, Flye, Canu, and Unicycler long-read-only on this dataset (Table 2). Since this strain diverged significantly from the published genome [38] by resequencing analysis (data not shown), we cannot use QUAST to evaluate the assembly qualities. We introduced two metrics: number of supplementary alignments and number of supplementary clusters. They indicate that there is alignment ambiguity due to structural errors based on the fact that we do not expect to see supplementary alignments or clusters in error-free assemblies.

**Table 2:** Evaluation of assembly on ONT sequences

Genome	Asm	# ctg	Length	Supl. A	Supl. C	M.R	Time (min)	Memory (G)	# Mapped PCR	Indels	SNV
*M.arginini*	B-assembler L	1	681,050	703	0	88.62	88	11.5	68	35	16
	B-assembler H	1	685,432	689	0	88.65	1294	23.8	70	7	16
	wtdbg2	1	660,247	2,806	12	86.92	7	2.1	57	84	156
	Flye	3	700,499	958	1	87.93	29	11.1	64	36	60
	Canu	1	793,572	873	1	87.89	2,883	15.7	65	39	41
	Unicycler L	2	755,568	759	2	87.91	73	10.6	66	42	99
	Unicycler H	61	407,877	8,646	10	76.43	1410	24.5	68	12	50
*M.amphoriforme*	B-assembler L	1	1,047,044	1,290	0	98.95	86	15.4	/	/	/
	wtdbg2	1	1,014,839	3,089	7	98.74	10	3.2	/	/	/
	Flye	1	1,052,944	2,218	1	98.7	254	9.1	/	/	/
	Canu	1	1,066,644	1,719	0	98.92	2,933	28.8	/	/	/
	Unicycler L	1	1,066,967	2,122	1	98.81	97	17.9	/	/	/

*# ctg* number of assembled contigs, *Suppl. A* number of Supplementary Alignments, *Suppl. C* number of Supplementary alignment Clusters, *M.R.* Mapping Rate, *B-assembler L., Unicycler L* the long-read modes of B-assembler and Unicycler, *B-assembler H. and Unicycler H.* the hybrid-read modes of B-assembler and Unicycler

As shown in Table 2, all the assemblers produced one complete contig from *M.amphoriforme*’s ONT sequencing data. However, B-assembler outperformed the other assemblers by generating a minimum number of supplementary alignments which were scattered all over the assembly and did not aggregate, while wtdbg2, and Unicycler at least one supplementary alignment cluster. In addition, B-assembler had a more uniform depth distribution when aligning the raw reads back to the assembled genome sequence (Additional file 1 Fig S3), while wtdbg2, Canu, and Unicycler dropped at two ends, and these depths were not consistent with the middle regions (Additional file 1 Fig S3). This suggests that it is challenging to forming a circular genome for these assemblers. This demonstrates that B-assembler outperforms the other tools for long-read-only real ONT data *de novo* assembly.

### Performance of B-assembler’s hybrid-read mode on real datasets

In order to demonstrate the performance of B-assembler in hybrid-read assembly, we isolated an *M. arginini* strain and deep sequenced on both Illumina MiSeq and Oxford MinION platforms. A total of 20,978 ONT reads (208X) and 2.8 M 150 bp × 2 paired-end Illumina reads (7900X) were generated. Over 78% of ONT reads are longer than 10kbp. We ran B-assembler and Unicycler on the hybrid data and all the other benchmarked algorithms on the long reads data.

As shown in Table 2, similar to the *M. amphoriforme* genome assembly, B-assembler got the least number of supplementary alignments and no supplementary clusters, while all the other tools generated at least 1 supplementary cluster and more supplementary alignments. This suggests that this genome contains regions hard to assemble for the other assemblers. The fact that Flye and Unicycler generated more than 1 contig as shown in Additional file 1 Fig S4 also supports this.

Besides B-assembler, Unicycler is a popular tool that supports hybrid data. B-assembler hybrid-read-mode generated one complete contig, while Unicycler yielded 61 contigs, the longest of which is 407,877 bp, much smaller than the majority, which average around 700kbp. In addition, there are 10 supplementary clusters and only 76.43% of the raw reads can be mapped to Unicycler’s hybrid assemblies. It is evident that the Unicycler’s hybrid-read mode is far from optimal in this case.

To further evaluate the accuracy of B-assembler, we used PCR amplification on 76 selected low-complexity locations and performed Sanger sequencing to get the sequences of these PCR amplifications. We mapped the amplicon sequences to individual assembly using minimap2. The mappable PCR sequences were defined as those with over 90% of their sequences that can be aligned to the assemblies and with a mapping quality of 60. We also calculated the differences (indels and mismatches) between the PCR amplicons and the contigs. For the total aligned PCR sequences, B-assembler had the minimum number of mismatches and indels. We further checked the locations of the errors and found that they tended to cluster at the ends of the PCR sequences, where Sanger sequencing may be inaccurate. This suggests that B-assembler can achieve a more accurate genome assembly which is critical for downstream analysis such as gene annotation. Therefore, the performance of B-assembler on *M. arginini* is better than other assemblers by generating a circular genome free of structural errors and with minimal base errors.

### Performance of B-assembler’s Long-read-only mode on PacBio sequence

PacBio sequencing data has a different error profile compared to Nanopore sequencing data. All the benchmarked assemblers can be applied to PacBio data. Therefore, we downloaded 14 bacterial PacBio sequencing data from the National Collection of Type Cultures (NCTC) 3000 project [39] and ran all the assemblers on this dataset. The NCTC also provided assembled references for these strains that were generated both automatically and manually. Thus, we used QUAST to evaluate the performance of these assemblers on this dataset. Detailed QUAST assembly metrics can be found in Additional file 1 Table S4.

Compared to the other assemblers, B-assembler achieved the least number of contigs (Table 3) and the N50s are also very close to the references (Additional file 1 Table S4). This suggests that B-assembler tends to generate complete genomes. Although overall B-assembler can get the minimum number of contigs, we did observe that species NCTC3610 and NCTC13348 produced more contigs than expected. This may be due to relatively poor data quality, and, as shown in the NCTC website, they are still pending, which means the genomes are yet to be assembled. B-assembler also demonstrated the best overall performance in resolving genome duplication sequences (“dup.” in Table 3). In addition, B-assembler also ranked in the first place in terms of generating the least number of misassemblies (“mis.” in Table 3).

**Table 3:** Selected QUAST statistics on the PacBio data

species ID	ref	B-assembler			Canu			Unicycler			Flye		
	#ctg	# ctg	dup	mis	# ctg	dup	mis	# ctg	dup	mis	# ctg	dup	mis
**NCTC13251**	1	**1**	1.004	**16**	1	1.006	**16**	1	1.006	**16**	1	**1.004**	**16**
**NCTC13349**	2	**2**	**1.001**	**0**	**2**	**1.001**	**0**	**2**	**1.001**	**0**	**2**	**1.001**	**0**
**NCTC13360**	9	**9**	**1.001**	**8**	15	1.038	20	11	1.016	13	10	1.014	10
**NCTC13307**	11	**10**	**1.001**	6	11	1.013	**2**	41	1.007	7	11	1.036	13
**NCTC3610**	19	**108**	**1**	**3**	204	1.032	20	297	1.054	5	120	1.005	6
**NCTC10005**	3	**4**	**1.002**	**10**	10	1.023	19	**4**	1.006	15	7	1.019	23
**NCTC13348**	2	14	**1.002**	**1**	19	1.015	11	25	1.004	3	**6**	1.02	7
**NCTC13616**	1	**1**	**1**	2	4	1.006	1	**1**	1.002	1	**1**	**1**	**0**
**NCTC13277**	1	**1**	**1**	2	3	1.004	2	**1**	1.001	**0**	**1**	**1**	**0**
**NCTC11192**	2	**1**	**1.002**	**1**	8	1.006	3	5	1.003	5	2	**1.002**	3
**NCTC13626**	2	**1**	**1**	2	4	1.006	2	3	1.001	**0**	**1**	**1**	**0**
**NCTC10963**	4	**1**	**1.001**	**1**	2	1.004	2	2	**1.001**	2	2	**1.001**	2
**NCTC12419**	1	**1**	1.001	2	**1**	1.003	**0**	**1**	1.001	**0**	**1**	**1**	**0**
**NCTC10833**	3	**2**	1.05	4	4	**1.01**	4	7	1.025	**3**	6	1.051	8

*# ctg* number of assembled contigs, *dup.* duplication ratio, *mis.* misassemblies larger than 1kbp

**Fig. 2:**
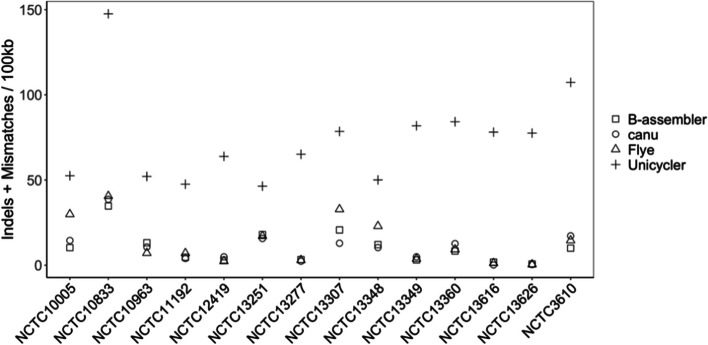
Indels and mismatches produced by the benchmarked assemblers on the 14 NCTC PacBio samples. The number of indels and mismatches were added per 100kbp

We also evaluated the accuracy of the assemblies based on QUAST’s metrics of number of mismatches per 100kbp and number of indels per 100kbp by comparing with the references. As shown in Fig. 2, the bacterial genome assembler, Unicycler, had the highest number of indels and mismatches, while Flye, Canu, and B-assembler were very close. These results indicate that B-assembler is also capable of assembling PacBio bacterial genomes with less base errors.

## Discussion

In this work, we present a new software package, B-assembler, for circular bacterial genome de novo assembly. B-assembler adopts a two-round assembly strategy, where the initial assembly is to build up the draft genome and reassembly ensures the formation of the circular genome. This strategy as well as its error correction modules guarantee an accurate genome assembly result. B-assembler supports both long-read-only and hybrid *de novo* assembly.

Benchmarked on simulation data, real sequencing data, and data from different long-read and hybrid platforms, B-assembler outperformed the other assemblers in terms of resolving structural errors, reducing base errors and indel errors, and generating a circularized genome at the same time. This is extremely critical to both basic and clinical research in the microbiological field.

B-assembler’s primary use is in cases where a researcher wishes to complete a bacterial assembly with a high quality in a short time. To facilitate this, future development of B-assembler will focus on speeding up the process by employing multithreading techniques. This will allow the user to efficiently get the assembly results. We will also extensively test alternative approaches in order to improve the accuracy for the hybrid dataset.

## Conclusions

B-assembler performed well on both short-read-only mode and hybrid-read mode, producing complete contigs than other assemblers. Perhaps more importantly, B-assembler produced fewer misassemblies than other assemblers. As Next-generation sequencing becomes more common, so will complete genome assemblies, enabling new research into genome structure. High-quality assemblies free of structural errors, such as those produced by B-assembler, will be critical to research in this field.

### Availability and requirements

*Project name*: B-assembler: a circular bacterial genome assembler;

*Project home page*: https://github.com/ChongLab/B-assembler;

*Operating system(s)*: Linux;

*Programming language*: python, shell;

Other requirements: python 3.0 or higher, and snakemake (see GitHub page);

License: MIT license;

Any restrictions to use by non-academics: terms stated in MIT License.

## Supplementary Information

**Additional file 1.** Supplementary text, tables and figures supporting the main text.

## Data Availability

The PacBio raw reads were downloaded from the European Nucleotide Archive (https://www.ebi.ac.uk/ena/browser/home). The datasets together with the following accession numbers: Bacillus (NCTC3610), accession no. ERR581147 and ERR581145; Enterobacter (NCTC10005), accession no. ERR688913and ERR688954; Staphylococcus (NCTC10833), accession no. ERR879369; Yersinia (NCTC10963), accession no. ERR710263; Legionella (NCTC11192), accession no. ERR832407; Salmonella (NCTC12419), accession no. ERR657651 and ERR657671; Bordetella (NCTC13251), accession no. ERR768071; Staphylococcus (NCTC13277), accession no. ERR879377; Clostridium (NCTC13307), accession no. ERR550486, ERR550480 and ERR581143; Salmonella (NCTC13348), accession no. ERR550498and ERR550489; Salmonella (NCTC13349), accession no. ERR772449); Staphylococcus (NCTC13360), accession no. ERR879378; Staphylococcus (NCTC13616), accession no. ERR879380 and ERR902071); Staphylococcus (NCTC13626), accession no. ERR879381 and ERR902070.

## Abbreviations

TGS: Third-Generation Sequencing
NGS: Next-Generation Sequencing
PacBio: Pacific Biosciences
ONT: Oxford Nanopore Technologies
M. arginini: Mycoplasma arginini
M. amphoriforme: Mycoplasma amphoriforme
GC: Guanine-cytosine
NCTC: National Collection of Type Cultures
