# Supplementary material for: B-assembler: a circular bacterial genome assembler
Source: BMC Genomics. 2022 May 11;23(Suppl 4):361. doi: 10.1186/s12864-022-08577-7 (PMC9092672; doi:10.1186/s12864-022-08577-7)
Supplement: Supplementary file 1 — Additional file 1. Supplementary text, tables and figures supporting the main text. [file 12864_2022_8577_MOESM1_ESM.docx]

**Additional file 1**

to the paper

“B-assembler - a circular bacterial genome assembler”

Fengyuan Huang^1,3^, Li Xiao^2^, Min Gao^1,2^, Ethan J Vallely^1^, Kevin Dybvig^3,4^,

T. Prescott Atkinson^4^, Ken B. Waites^5^, Zechen Chong^1,3, *^

^1^Informatics Institute, Heersink School of Medicine, the University of Alabama at Birmingham, Birmingham, Alabama, 35294, United States of America; ^2^Department of Medicine, Heersink School of Medicine, the University of Alabama at Birmingham, Birmingham, Alabama, 35294, United States of America; ^3^Department of Genetics, Heersink School of Medicine, the University of Alabama at Birmingham, Birmingham, Alabama, 35294, United States of America; ^4^Department of Pediatrics, Heersink School of Medicine, the University of Alabama at Birmingham, Birmingham, Alabama, 35233, United States of America; ^5^Department of Pathology, Heersink School of Medicine, the University of Alabama at Birmingham, Birmingham, Alabama, 35233, United States of America

^*^Correspondence: [zchong@uab.edu](mailto:zchong@uab.edu)

**Note 1. Comparison of contiguity of assemblies with different coverages**

B-assembler does not consider all varying-length reads; it selects parts of the top sorted reads by their length for the initial assembly. To decide which part of the reads will be selected to directly make the initial assembly, we sorted the simulation sequences by their lengths and extracted the top long-reads with several levels (10x; 20x; 30x; 50x; 70x; 100x) of coverage. These subsets of reads were then applied to B-assembler to generate the initial assembly. The contiguity of the assemblies produced by subsets reads is relatively close to the coverage. The subsets of reads of more than 50x had the capability to generate complete contigs. Given relatively high coverage of bacteria long-read sequencing, B-assembler considers 50x as acceptable for our pipeline.

**Note 2. Data set and reference genomes**

In the computational experiment, we used 16 bacterial species of simulation and real data sets from Oxford Nanopore, PacBio, and Illumina paired-end sequencing. The simulation sequence of Oxford Nanopore and Illumina pair-end reads were from *M.arginini*, whose reference is HAZ 145_1. We used QUAST to evaluate the performance of B-assembler on simulation sequence and compared to other assemblies. We had real ONT sequence from two mycoplasma strains *M.arginini* and *M.amphoriforme* which were isolated from a patient and a cow. These two strains were quite different from the published genome. For example, their hosts and their pathogenicity totally differed from their references. Therefore, we did not use QUAST, which needs a reference genome, to demonstrate the quality of assembly results. We downloaded the PacBio sequence and reference genomes of 14 bacterial strains from the European Nucleotide Archive. Links to the genome reference files are specified below:

1. *M.arginini*

genome: <https://www.ncbi.nlm.nih.gov/nuccore/AP014657.1?report=fasta>

1. 14 bacterial genome references of PacBio sequence:

genome: <ftp://ftp.sanger.ac.uk/pub/pathogens/circlator/Supplementary_data/Circlator_supplementary_data.tar.gz>


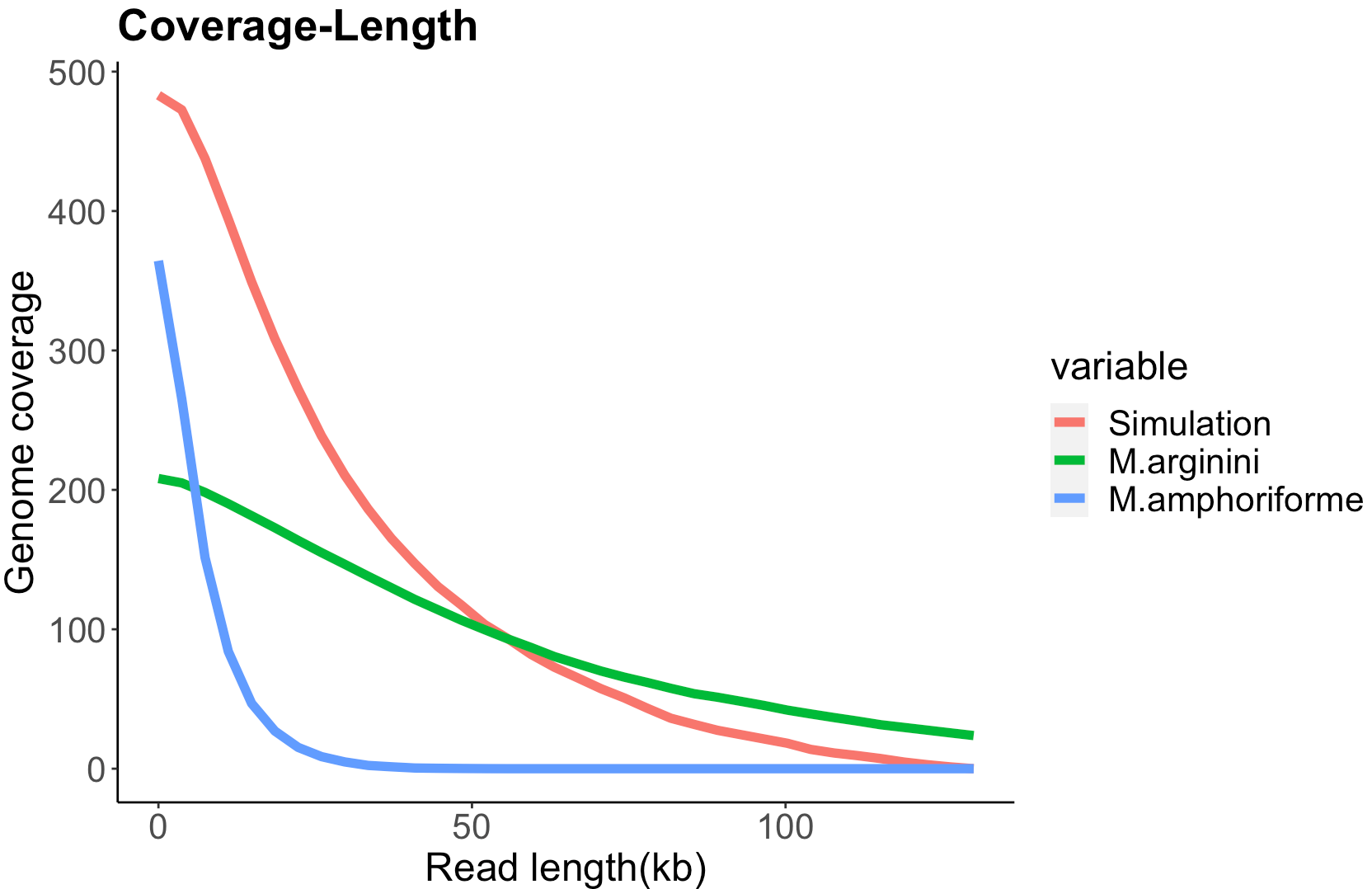


**Fig S1.** The read lengths and their associated coverage for the simulation ONT, real *M.arginini* and *M.amphoriforme* ONT reads. The Y axis is genome coverage of reads (reads >length). The X axis is different read length.

A.


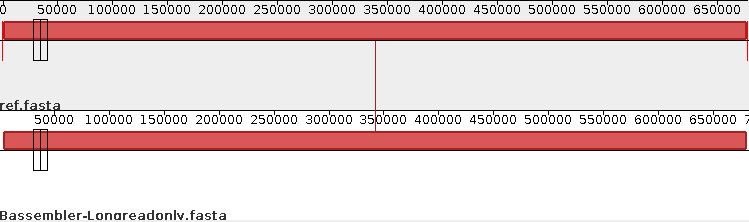


B.


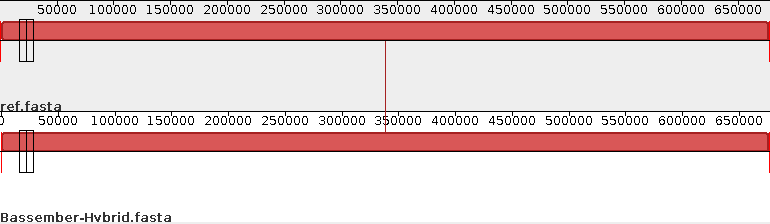


C.


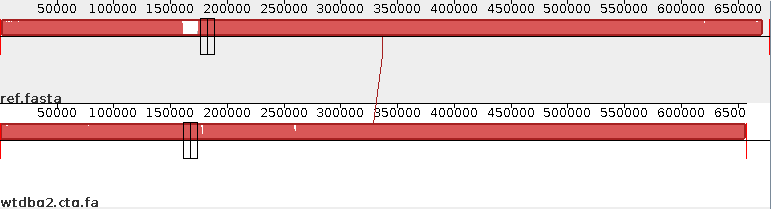


D.


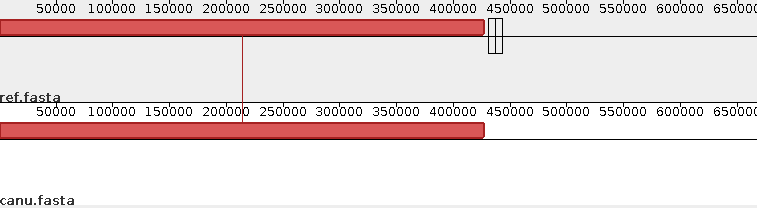


E.


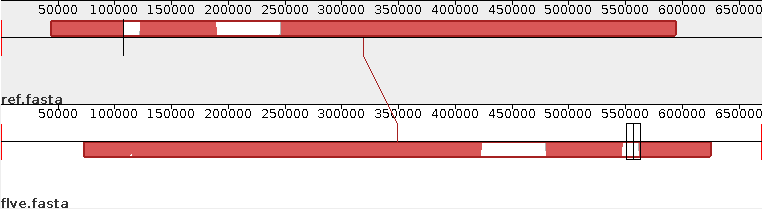


F.


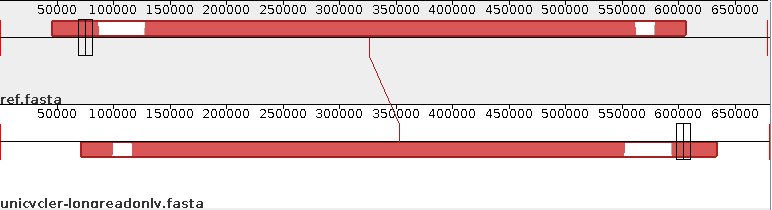


G.


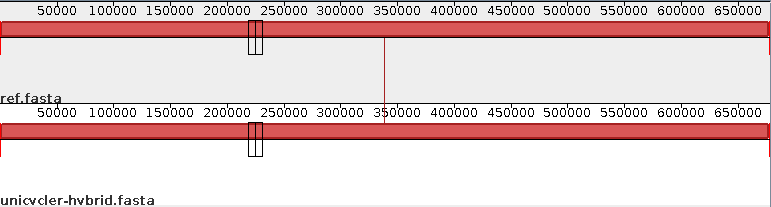


**Fig S2.** Mauve Genome alignments of reference and individual assembly generated with the simulated ONT sequences. The order of alignments, from top to bottom, is A: B-assembler long-read only mode assembly. B: B-assembler hybrid mode assembly. C: wtdbg2 assembly. D: canu assembly. E: Flye assembly. F: Unicycler long-read only mode assembly. G: Unicycler hybrid mode assembly. Each row is a genome. In each panel, the first row is the reference genome. Each red block is genetically similar, and each white black is unique sequence (unaligned sequence). Blocks on the top row are in the same orientation, while blocks on the bottom row are in reverse orientation. The red line between two rows is the relative alignment of blocks.

A.

B.


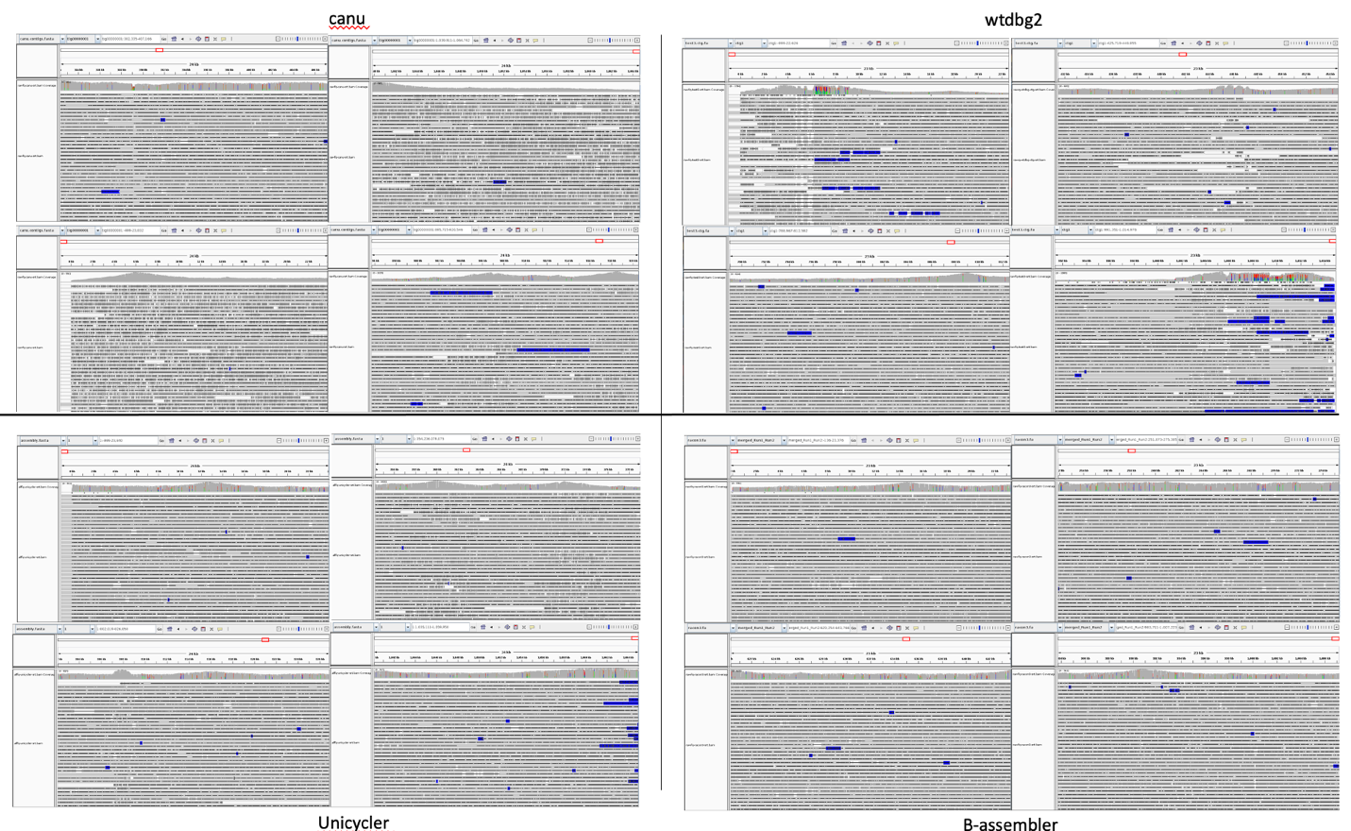


1

2

3

C.

D.

**Fig S3.** IGV view of *M.amphoriforme* ONT reads alignment. Four IGV views were selected for each assembly alignment. The order is: A: canu; B: wtdbg2; C: Unicycler; D: B-assembler. In each IGV view, the frames from top to bottom are shown as dash line frame is 1: genomic coordinates, 2: long reads depths tracks, and 3: reads alignments. In read alignments box, blue lines are supplementary alignments. Red marked box is the area that has a structural error.

A


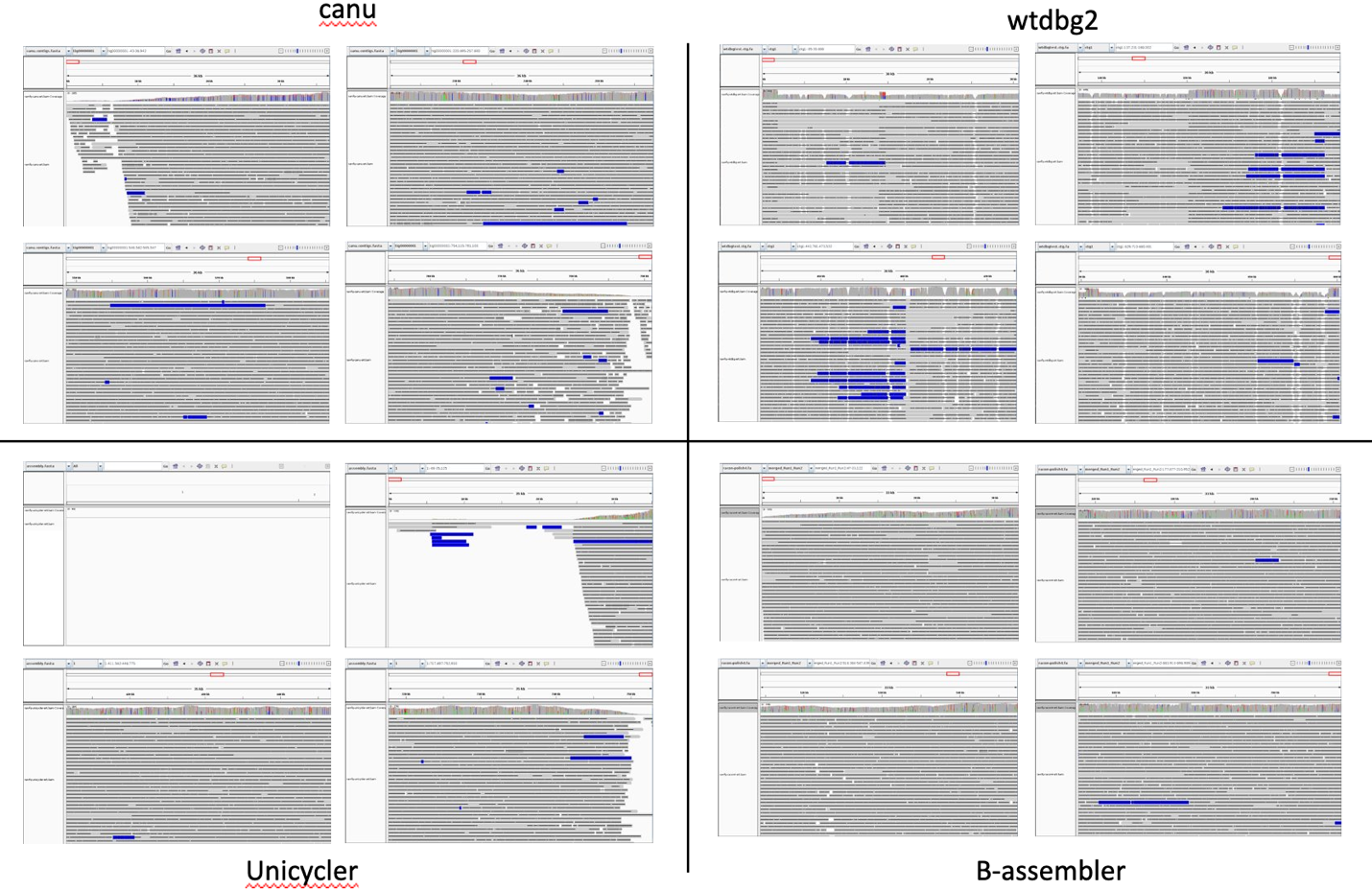


B


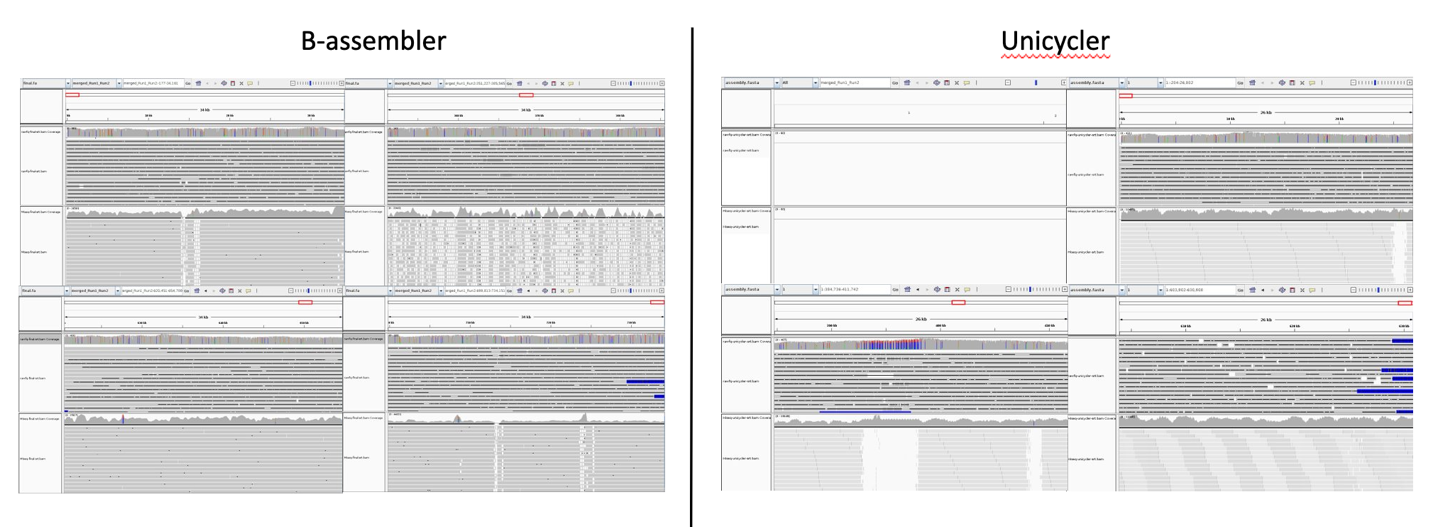


1

2

3

4

5

**Fig S4.** IGV view of *M.arginini* sequence alignment. A: IGV view of ONT read alignment of assemblies from long-read only mode. B: IGV view of ONT and Illumina read alignment of assemblies from B-assembler and Unicycler’s hybrid mode. In B, the frame from top to bottom is 1: genomic coordinates, 2: Long reads depths tracks, 3: Long read alignments. 4: Short read depth tracks and 5: Short read alignment. In the read alignment box, the blue lines are the supplementary alignments. The red marked boxes are structural errors.

**Table S1**. Contiguity of assemblies generated by subsets of longest simulation ONT reads.


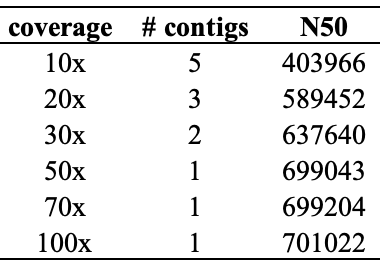


Note: The number of contigs and N50 of contigs generated by the initial step of B-assembler’s long-read only mode with subsets of ONT reads. The coverage is the longest simulation ONT reads with depth range from 10x to 100x; #contigs is the number of contigs generated by the initial assembly of B-assembler’s long-read only mode. N50, length of a contig, such that all the contigs of at least the same length altogether cover at least 50% of the assembly, describes the “completeness” of the assemblies.

**Table S2.** Summary of QUAST result with simulation ONT data.
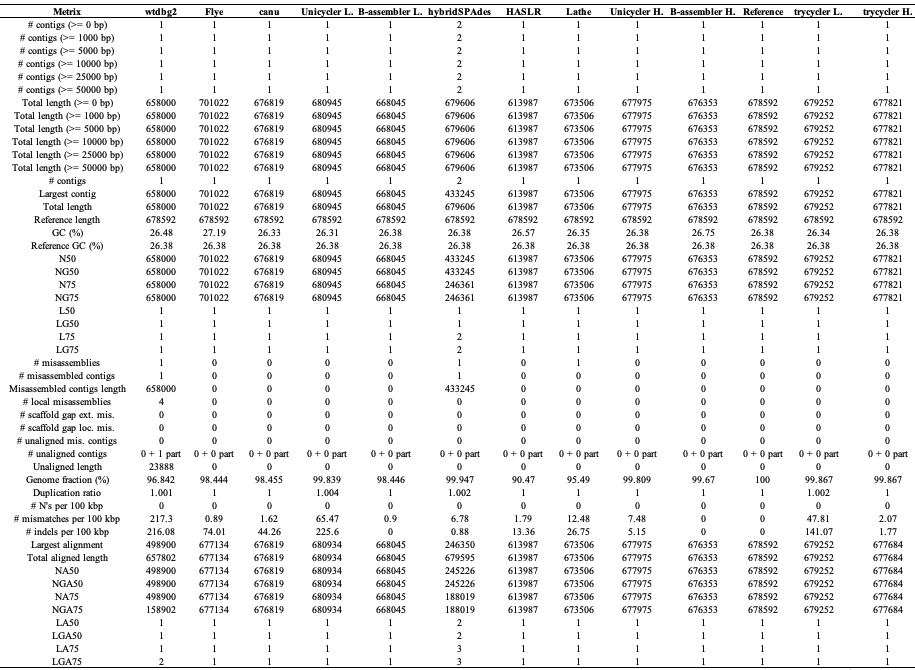


Note: All QUAST metrics generated by input from wtdbg2, Flye, canu, Unicycler Long-read mode, hybridSPAdes, HASLR, lathe, B-assembler long-read mode, Unicycler hybrid-read mode,

B-assembler hybrid-read mode, trycycler Long-read mode, trycycler hybrid-read mode and reference genome.

**Table S3**. Comparison of short read polishing tools.


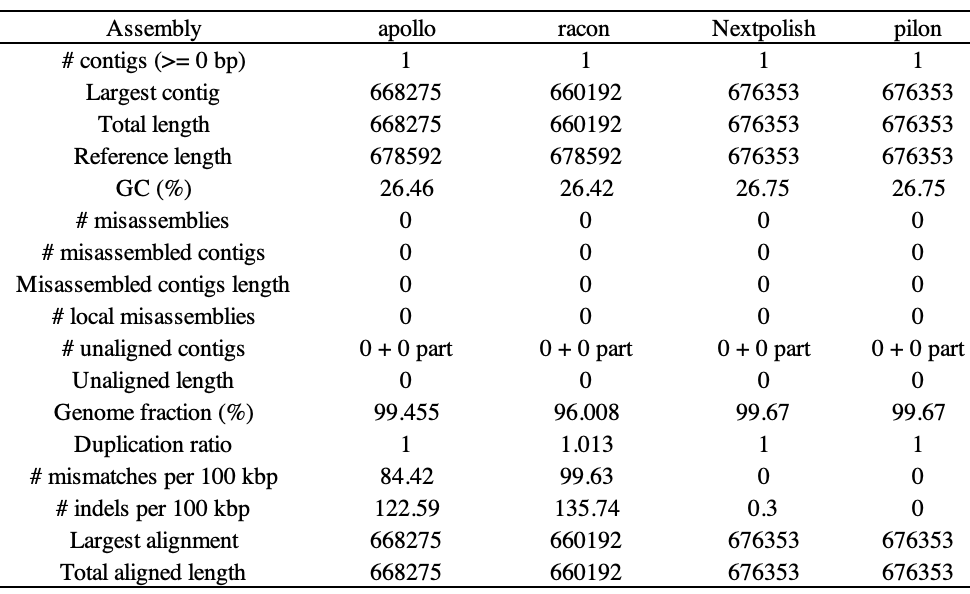


Note: apollo, racon, Nextpolish and pilon were applied to B-assembler’s hybrid mode. Simulation ONT and Illumina sequences were used for testing the performance of these tools. Evaluations was generated by QUAST.

**Table S4.** Summary of each component of B-assembler.

**
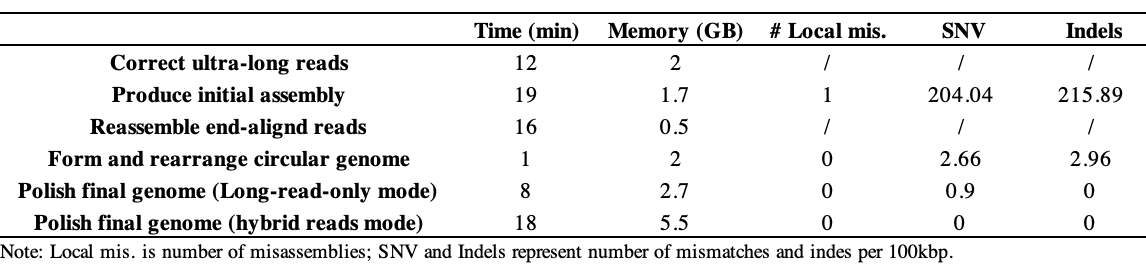
**

**Table S5.** Summary of QUAST result with 14 bacterial species.


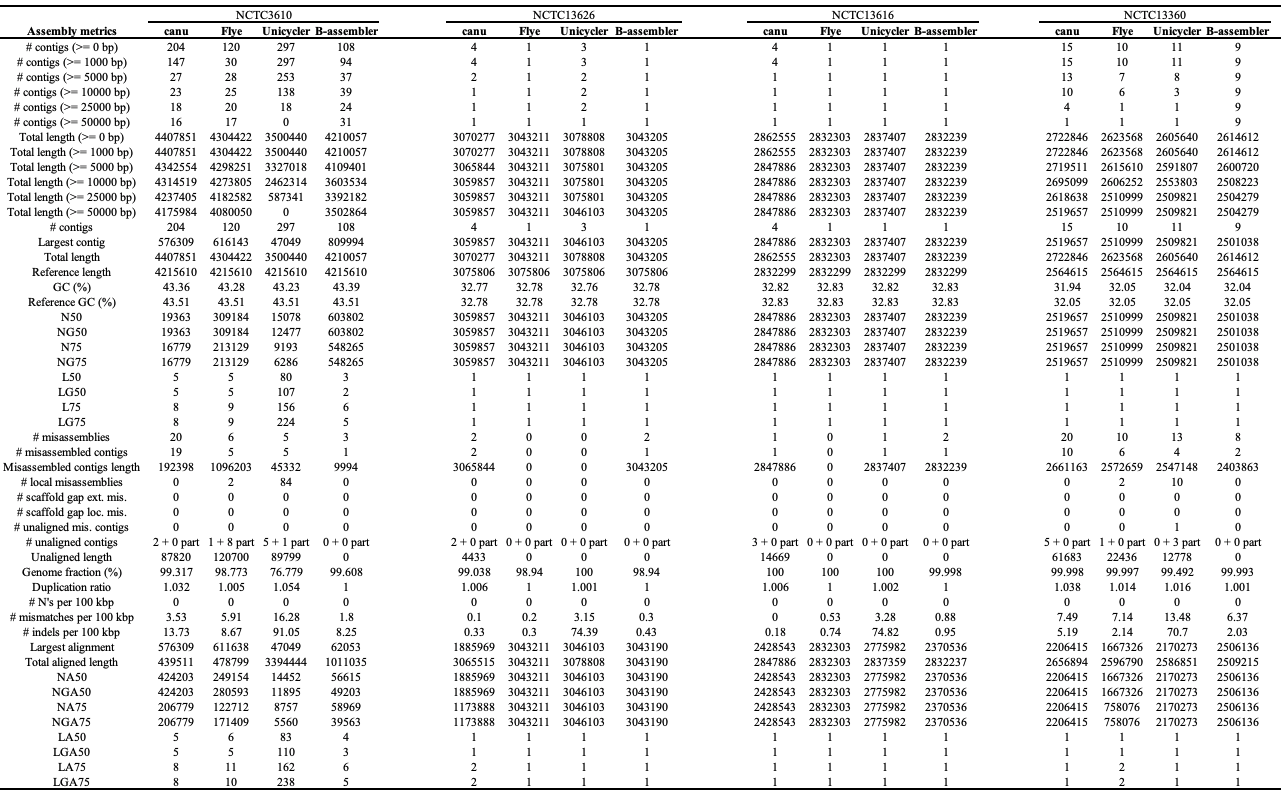


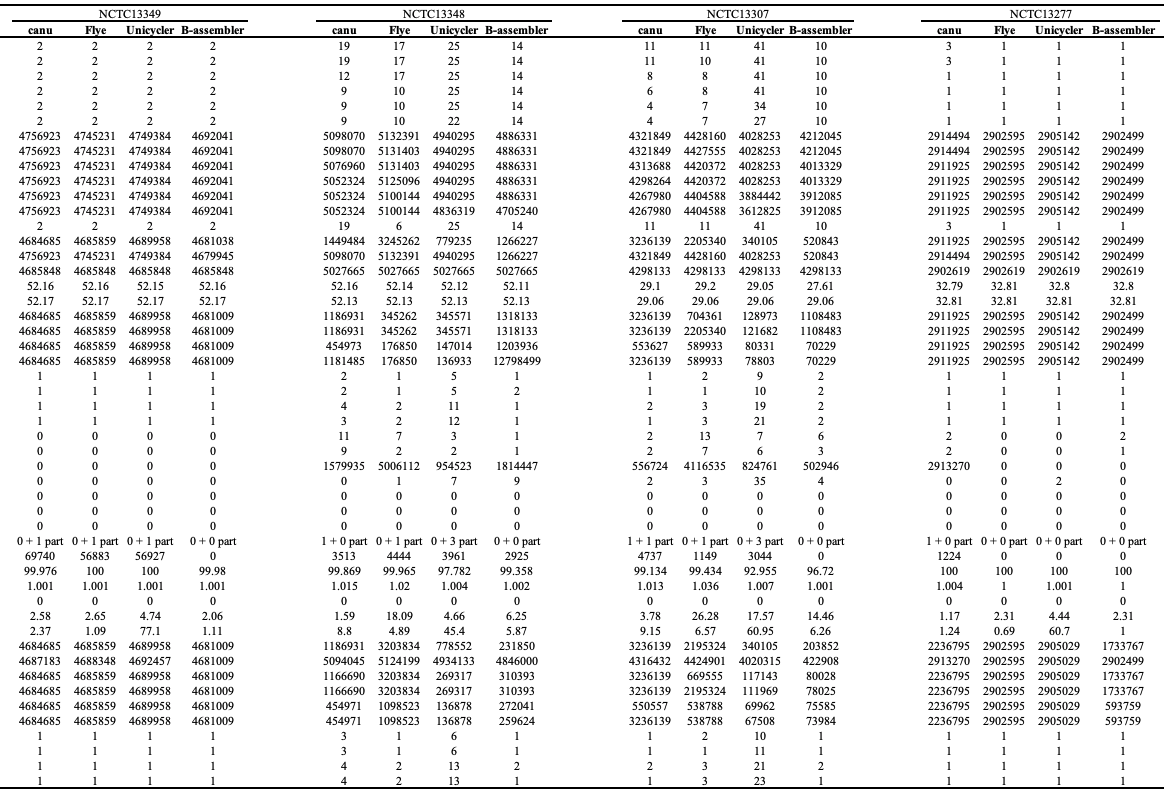


Note: All metrics from QUAST are generated by input from wtdbg2, Flye, canu, Unicycler, B-assembler for each bacterial species.

**Table S6.**  Summary of 14 bacterial species had PacBio sequence.


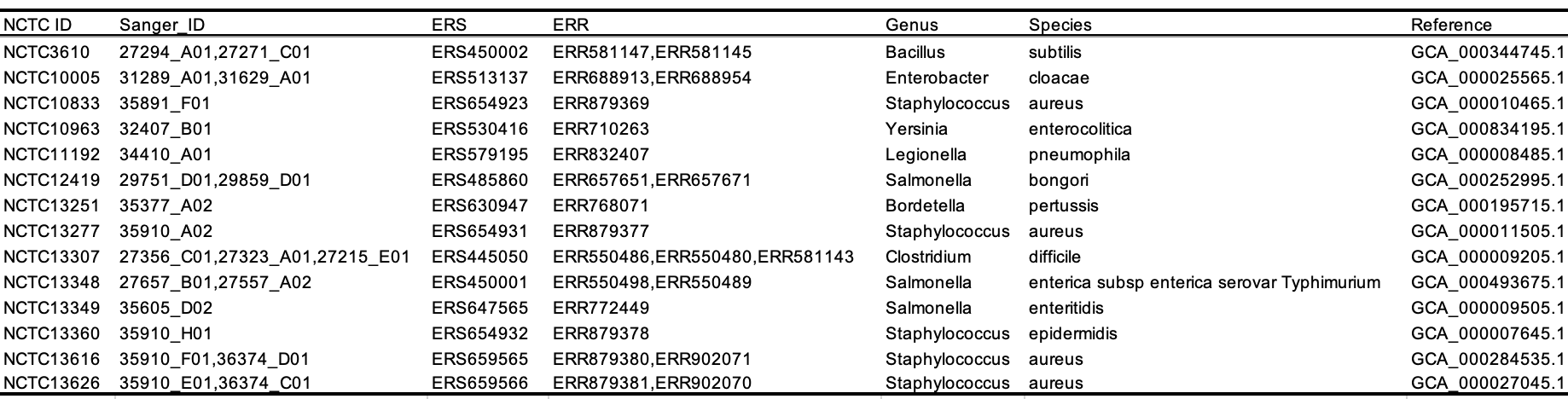


Note: NCTC ID, Sanger ID, ERS database ID, ERR database ID, Genus, Species and reference of 14 bacterial strains.

1. Bankevich A, Nurk S, Antipov D, Gurevich AA, Dvorkin M, Kulikov AS, Lesin VM, Nikolenko SI, Pham S, Prjibelski AD *et al*: **SPAdes: a new genome assembly algorithm and its applications to single-cell sequencing**. *J Comput Biol* 2012, **19**(5):455-477.
